# Therapeutic potential of natural products in schistosomiasis-associated liver fibrosis

**DOI:** 10.3389/fphar.2024.1332027

**Published:** 2024-05-06

**Authors:** Cuiling Liu, David Fisher, Khrystyna Pronyuk, Erkin Musabaev, Nguyen Thi Thu Hien, Yiping Dang, Lei Zhao

**Affiliations:** ^1^ Department of Infectious Diseases, Union Hospital, Tongji Medical College, Huazhong University of Science and Technology, Wuhan, China; ^2^ Department of Medical Biosciences, Faculty of Natural Sciences, University of the Western Cape, Bellville, South Africa; ^3^ Infectious Diseases Department, O.Bogomolets National Medical University, Kyiv, Ukraine; ^4^ The Research Institute of Virology, Ministry of Health, Tashkent, Uzbekistan; ^5^ Hai Phong University of Medicine and Pharmacy, Hai Phong, Vietnam; ^6^ Department of Vascular Surgery, Union Hospital, Tongji Medical College, Huazhong University of Science and Technology, Wuhan, China

**Keywords:** natural products, plant extracts, schistosomiasis, liver disease, fibrosis

## Abstract

Schistosomiasis is a parasitic disease that endangers human health and social development. The granulomatous reaction of Schistosoma eggs in the liver is the main cause of hepatosplenomegaly and fibrotic lesions. Anti liver fibrosis therapy is crucial for patients with chronic schistosomiasis. Although Praziquantel is the only clinical drug used, it is limited in insecticide treatment and has a long-term large-scale use, which is forcing the search for cost-effective alternatives. Previous research has demonstrated that plant metabolites and extracts have effective therapeutic effects on liver fibrosis associated with schistosomiasis. This paper summarizes the mechanisms of action of metabolites and some plant extracts in alleviating schistosomiasis-associated liver fibrosis. The analysis was conducted using databases such as PubMed, Google Scholar, and China National Knowledge Infrastructure (CNKI) databases. Some plant metabolites and extracts ameliorate liver fibrosis by targeting multiple signaling pathways, including reducing inflammatory infiltration, oxidative stress, inhibiting alternate macrophage activation, suppressing hepatic stellate cell activation, and reducing worm egg load. Natural products improve liver fibrosis associated with schistosomiasis, but further research is needed to elucidate the effectiveness of natural products in treating liver fibrosis caused by schistosomiasis, as there is no reported data from clinical trials in the literature.

## 1 Introduction

Schistosomiasis is a parasitic disease, in which trematodes infections poses a serious threat to human health and social development. Schistosomiasis is found in 78 countries in the tropics and subtropics and is predominantly endemic in sub-Saharan Africa (WHO, 2022). Intestinal schistosomiasis and urogenital schistosomiasis are two primary pathologies caused by Schistosoma infections in humans, with the former being primarily caused by *S. japonicum* and *S. mansoni* and the latter by *S. haematobium*. (McManus et al., 2018). Worms parasitize the veins of human hosts to mate and lay eggs are excreted in feces or urine. These eggs hatch as miracidia and infect intermediate species-specific host snails in fresh water. After 4–6 weeks, the eggs develop into infectious cercariae that can penetrate human skin and cause disease. Acute infections occur mostly in locals, travelers and immigrants and present symptoms of transient urticaria rash, allergic pneumonia, and Katayama syndrome. (Clerinx and Van Gompel, 2011). The progression of these infections can be characterized by chronic abdominal pain, loss of appetite, and liver polyps, and eventually lead to hepatosplenomegaly, portal hypertension, ascites, gastrointestinal varices, and even life-threatening gastrointestinal bleeding (Colley et al., 2014). Currently, the only clinically effective drug for treating schistosomiasis is praziquantel (Kabuyaya et al., 2023). However, due to the incomplete efficacy of praziquantel and the potential for drug resistance, there is an urgent need to find cost-effective alternatives or complementary treatments (Santana et al., 2021). More research is crucial to investigate novel targeting mechanisms of schistosome-induced liver fibrosis in order to discover new drugs or praziquantel analogs that can treat schistosomiasis.

## 2 Pathogenesis of chronic schistosomiasis

Fibrosis of the liver and portal system is the main pathological manifestation of intestinal schistosomiasis and is the result of an immune response caused by the invasion of schistosome eggs into the liver and blood vessels. The deposition of eggs causes a granulomatous inflammatory response mediated by CD4 T lymphocytes^+^, which is characterized by a markedly active Th2 immune response, such as increased levels of cytokines and chemokines, and the recruitment of lymphocytes, neutrophils, eosinophils, macrophages, and fibroblasts, followed by extracellular matrix (ECM) and collagen fibril production of liver tissue, with the eggs being trapped in the liver and unable to be excreted, finally resulting in a fibrotic inflammatory infiltrate forming around the eggs (Chiu et al., 2003; Amaral et al., 2017; Ho et al., 2022). The expedited Th2 response during the schistosomiasis infection may be related to alternate activation of macrophages. During the chronic infection phase, alternatively activated macrophages (M2 macrophages) are stimulated by IL-13, IL-33, IL-4, and ROS to regulate the expression of Arg1, IL-10, and TGF-β1, which act directly or indirectly on hepatic stellate cells and contribute to α-SMA and collagen production, leading to liver fibrosis (Peng et al., 2017; Tan et al., 2018; Yu et al., 2021). The soluble egg antigen (SEA) can also activate M2 macrophages via STAT6 and PI3K signaling pathways or directly activate hepatic stellate cells via the P38/JNK MAPK signaling pathway (Liu P. et al., 2013; Tang et al., 2017). In addition, the deposition of eggs significantly reduced the enzymatic activities of O_2_^.-^ and H_2_O_2_ detoxification, by superoxide dismutase (SOD), catalase, and glutathione peroxidase (GSHPx) and increased the levels of hepatic products from lipid peroxidation, which may stimulate the progression of liver fibrosis (Gharib et al., 1999).

## 3 Natural products against schistosomiasis-associated liver fibrosis

An increasing number of studies have demonstrated that bioactive ingredients of medicinal plants are a promising alternative to current clinical therapy. The current direction of anti-schistosomiasis drug research is focused on the screening of compounds with therapeutic targets and the development of praziquantel analogs. Anti-fibrotic treatment is essential for patients with chronic schistosomiasis as even deworming does not completely stop the progression of liver fibrosis (Bergquist et al., 2017; LoVerde et al., 2021). In schistosomiasis-endemic countries such as China, Brazil, Zimbabwe, and Kenya, the anti-schistosomiasis pharmacological effects of natural products or plant extracts have been extensively studied in an attempt to discover alternative drugs (Molgaard et al., 2001). Currently, the active mechanism of various natural products in the treatment of schistosomiasis-associated liver fibrosis has been reported, which may modulate fibrotic factors such as IL-13, growth stimulation expressed gene 2 (ST2), TGF-β1, TNF-α and anti-fibrotic factors such as IL-10, Tregs, MHC II through intracellular signaling pathways such as NF-κB pathway, PI3K/AKT pathway and TGF-β1/Smad pathway (Liu et al., 2014; Tang et al., 2017; Kamdem et al., 2018; Huang et al., 2020).

### 3.1 Natural compounds

Based on the diversity that biological exploration of natural products provides for drug discovery, the active substances of natural products and their independent pharmacological effects in mixtures have attracted much attention (Phillipson, 2001; Simoben et al., 2018). Traditional medicinal plants are highly diverse and many metabolites have been shown to have therapeutic effects on liver fibrosis in schistosomiasis (Table 1).

**TABLE 1:** Natural compounds for schistosomiasis-associated liver fibrosis.

Natural compounds	Main source	Experimental model	Mechanisms	Efficacy	References
Artemisinin and its derivatives	*Artemisia annua L*	Mice by S. mansoni/S. japonicum	Inhibition of apoptosis; Affects female worms metabolism	Reduced worm and egg loads, reduced liver granulomas	Zhai et al. (2000), Abdin et al. (2013), El-Lakkany and Seif El-Din (2013), Madbouly et al. (2015), Hegazy et al. (2018)
Chlorogenic acid	Green coffee beans	Mice by S. japonicum; LX2 induced by TGF-β1/rIL-13	Regulating IL-13/miR-21/Smad7 and TGF-β1/Smad7 Signaling Pathway	Inhibition of HSCs proliferation; Inhibits collagen deposition	Wang et al. (2017), Yang et al. (2017)
Corilagin	*Phyllanthus niruri L*	Mice by S. japonicum	Regulating IL-13/miR-21/Smad Signaling Pathway; M2 macrophages polarization	Reduced expression of fibrosis factor; Reduced hepatic fibrosis and granuloma area	Yang et al. (2016), Li et al. (2017)
Resveratrol	Grape	Mice by S. mansoni/S. japonicum; HSC-T6 induced by TGF-β1	Reduced ROS accumulation and enhanced oxidase activity; SIRT1/NF-κB signaling pathway	Reduced ECM deposition; Suppression of inflammation	Soliman et al. (2017), Chen et al. (2019), Hao et al. (2022), Mostafa et al. (2023)
Curcumin	*Curcuma longa L*	Mice by S. mansoni/S. japonicum	Enhanced oxidase activity; Immunomodulation (NF-κB)	Reduced worm and egg loads, reduced liver granulomas; Inhibition of pro-fibrotic mediators	Jagetia and Aggarwal (2007), Allam, 2009; Chen et al. (2009), Abu Almaaty et al. (2021)
Genistein	*Glycine* max *(L.) Merr*	Mice by S. japonicum; HSCs induced by MφCM (stimulated by SEA)	SIRT1/TGFβ/Smad3 pathway	Suppression of inflammations; Inhibition of HSCs activity; Reduced liver granulomas	Wan et al. (2017), Zhou et al. (2021a)
Paeoniflorin	*Paeonia lactiflora Pall*	Mice by S. japonicum; HSCs induced by TGF-β1(PMCM stimulated by SEA)	TGFβ/Smad Signaling Pathway; Alternative activation of macrophages	Inhibition of HSCs proliferation; Inhibition of pro-fibrotic mediators	Chu et al. (2007), Li et al. (2010), Chu et al. (2011)

Abbreviations: S. mansoni/S. japonicum, Schistosoma mansoni/Schistosoma japonicum; IL-4/6/10/12/13, interleukin 4/6/10/12/13; TGF-β1, transforming growth factor beta 1; M2, alternatively activated macrophage; SEA, soluble egg antigen; MφCM, macrophage-conditioned medium; NF-κB, nuclear transcription factor-κB; SIRT1, sirtuin 1/silent mating type information regulation two homolog-1; HSC, hepatic stellate cell; PMCM, peritoneal macrophage-conditioned medium.

#### 3.1.1 Artemisinin and its derivatives

*Artemisinin* is a sesquiterpene lactone derived from *Artemisia annua L.*, and artemisinin-based combination therapies (ACTs) are widely used to treat malaria. *Artemisinin* including its derivatives such as *artesunate*, *dihydroartemisinin*, and *artemether* has also been shown to have pharmacological effects such as anti-cancer, anti-viral, anti-inflammatory, and anti-parasitic (Ho et al., 2014). *Artemisinin* and its derivatives have been shown to kill helminths in animal models (rabbits, rats, dogs) with worm reduction rates ranging from 41% to 98%, which is based on its high lethality to larvae and females (You et al., 1992; Xiao et al., 1994; Gold et al., 2017; Correa et al., 2019). In *Schistosoma mansoni* infected mice, the combination of artesunate (400 mg/kg) and praziquantel (500 mg/kg) significantly decreased hepatic P53 expression and increased Bcl-2 expression (Hegazy et al., 2018). Lower doses of artemether (50 mg/kg), artemether reduced the number of eggs in mice’s feces, whereas higher doses of 400 mg/kg artemether greatly reduced the number and diameter of liver granulomas (Lescano et al., 2004; El-Beshbishi et al., 2013). A possible mechanism for this reduction is the inhibition of the expression of schistosomal metabolic enzymes such as glycolytic key enzymes as well as thioredoxin glutathione reductase (TGR), cytochrome c peroxidase (CcP) and SOD (Zhai et al., 2000; Abdin et al., 2013; El-Lakkany and Seif El-Din, 2013). Furthermore artemether has been shown to mediate a shift from a Th2 to a Th1 response in schistosomes, characterized by an increase in IFN-γ levels and a decrease in IL-4 and IL-10 levels (Madbouly et al., 2015). Interestingly, Keiser et al. found that artemether did not rely on synergy with the immune response for its anti-schistosomal effects, even though immunomodulation was beneficial in suppressing egg-induced hepatotoxicity (Keiser et al., 2010). In summary, *artemisinin* and its derivatives may be a potential treatment for schistosomiasis liver fibrosis.

#### 3.1.2 Chlorogenic acid

*Chlorogenic acid*, 5-O-caffeoylquinic acid (5-CQA), a natural polyphenolic compound, is widely found in various fruits, vegetables, and medicinal plants, such as apples, eggplants, coffee beans, *Lonicera japonica* Thunb. And *Eucommia ulmoides Oliv*. It is most abundant in green coffee beans. It has significant protective effects on cardiovascular, gastrointestinal, liver, nerve, and metabolism due to its antioxidant, antibacterial, and anticancer biological activities (Naveed et al., 2018; Lu et al., 2020). *Chlorogenic acid* has been reported to protect against different types of liver fibrosis through NOX/ROS/MAPK, ERK/Nrf2, TLR4, and NF-κB pathways (Shi et al., 2013; Shi et al., 2016; Yuan et al., 2017; Wei et al., 2018). *Chlorogenic acid* inhibited the elevated expression of TGF-β receptor I, CTGF, and α-SMA after IL-13 treatment of LX2 cells in a dose-dependent manner, ranging from 56 μM to 225 µM. *In vivo* studies in *Schistosoma japonicum*-infected mice have shown that the use of *chlorogenic acid* (5–20 mg/kg for 4 weeks) reduces IL-13 expression and significantly reduces the size of liver granulomas (Wang et al., 2017). Further mechanisms suggest that IL-13 affects miR-21/Smad7 signaling, which in turn affects liver fibrosis. IL-13 is a key factor in the progression of schistosomiasis disease which is consistent with the view of Wynn et al. (2004). Furthermore, 56–225 µM *chlorogenic acid* also directly interferes with miR-21-regulated TGF-β1/Smad7 signaling, which reduces the expression of CTGF, TIMP and MMP-9 and decreases collagen and ECM deposition (Yang et al., 2017).

#### 3.1.3 Corilagin

*Corilagin*, β-1-O-galloyl-3,6-(R)-hexahydroxydiphenoyl-d-glucose, a natural ellagitannin, mainly derived from plants such as *Phyllanthus niruri L.* and *Geranium sibiricum L.*, has a variety of pharmacological activities including anti-cancer, anti-inflammatory, antioxidant and hepatoprotective (Li et al., 2018; Gupta et al., 2019). Polarization of M2 macrophages induced by IL-4/IL-13 plays an important role in the granuloma response of *Schistosoma* eggs (Herbert et al., 2004). *Corilagin* (20, 40, 80 mg/kg/day for 28 days) significantly reduced hepatic fibrosis by decreasing the expression of pro-fibrotic factors (e.g., IL-13, IL-13 receptor α1, IL-4 receptor α), as well as by decreasing the expression of PPARγ, KLF4, SOCS1, and p-STAT6, and by inhibiting the polarization of M2 macrophage in *Schistosoma* hepatic tissues in mice (Du et al., 2016). Li et al. showed that *Corilagin* (39–157 μM, 24 h) significantly inhibited downstream fibrotic factors by interfering with the binding of IL-13 to IL-13Rα1 in Ana-1 cells (Li et al., 2017). Yang et al. found that treatment of schistosome mice with 20 mg/kg Corilagin reduced the number of liver eggs and effectively protected against liver fibrosis by inhibiting miR21 regulation of Smad7 and Smad1/2 phosphorylation (Yang et al., 2016). The above studies show that *Corilagin* is an effective drug in the treatment of schistosomiasis-induced liver fibrosis.

#### 3.1.4 Resveratrol

*Resveratrol* (3,5,4 0-trihydroxy-trans-stilbene) is a non-flavonoid polyphenol found in over 70 plants such as *Veratrum grandiflorum (Maxim. ex Miq.) O. Loes.*, *Polygonum cuspidatum* Siebold & Zucc., grapes and peanuts. *Resveratrol* (RSV) is highly valued for its antioxidant, anti-inflammatory, anti-cancer, anti-diabetic, anti-aging, cardioprotective, and neuroprotective effects (Zhang L. X. et al., 2021). RSV-containing nanocarriers reduced ROS levels, inhibited the growth of activated HSC-T6 cells *in vitro* (20 µM) and significantly reduced hepatic ECM accumulation *in vivo* (5 mg) (Hao et al., 2022). The reduction of GSH and SOD expression in livers infected with schistosomes was significantly reversed in 2 weeks of treatment with 20 mg/kg RSV (Soliman et al., 2017). Chen et al. showed that RSV (400 mg/kg for 3 days) increased mitochondrial membrane potential (Δφm) and peroxisome proliferator-activated receptor-γ coactivator 1α (PGC-1α) expression in mouse liver. Interestingly, the improvement of mitochondrial function was not the only factor that affected liver fibrosis amelioration with RSV(Chen et al., 2019). As a Sirt-1 activator, RSV (20 mg/kg or 100 mg/kg for 4 weeks) reduced anti-inflammatory markers and anti-fibrotic markers in schistosome-infected mice via the SIRT1/NF-κB signaling pathway (Mostafa et al., 2023). In addition, RSV (20 mg/kg for 3 weeks) inhibited the development and progression of liver granulomas by regulating Th17/Treg responses (Han et al., 2019). Therefore, RSV may exert its anti-schistosomal liver fibrosis effects through the above mechanisms.

#### 3.1.5 Curcumin

*Curcumin* (1,7-bis-(4-hydroxy-3-methoxyphenyl)-hepta-1,6-diene-3,5-dione), is a natural polyphenolic compound, mainly extracted from the rhizome of *Curcuma longa L. Curcumin* is the key active component of turmeric, showing antioxidant, anti-inflammatory, anti-cancer, anti-microbial, and tissue (heart, nerve, liver) protective effects (Sohn et al., 2021; El-Saadony et al., 2022). The protective effects of curcumin against different types of liver injury are mainly mediated by reducing lipid peroxidation, activating the Nrf2 signal and inhibiting NF-κB activity (Khan et al., 2019). *Curcumin* (50–200 mg/kg) was found to upregulate MMP-1 and inhibit TIMP-1, resulting in a reduction in liver granuloma volume by up to 79% and collagen content by 38.6% (Li et al., 2007). Low expression of GSH, GST, SOD, and CAT caused by *S. mansoni* infection increased significantly after 2 weeks of treatment with 40 mg/kg *curcumin* (Abu Almaaty et al., 2021). A total dose of 400 mg/kg *curcumin* treatment was found to suppress serum levels of IL-12 and TNF-α in the infected group, possibly related to immune regulation triggered by inhibition of NF-κB activity (Jagetia and Aggarwal, 2007; Allam, 2009). Additionally, it significantly raised the mRNA expression of PPAR while reducing TGF-β1 (Chen et al., 2009). These studies suggest that *curcumin* may be an effective in the treatment of schistosomiasis-induced liver fibrosis.

#### 3.1.6 Genistein

*Genistein* (5,7-dihydroxy-3-(4-hydroxyphenyl) chromium-4-one) is the most potent functional component of soy (*Glycine* max *(L.) Merr.*) isoflavone products, with anti-cancer, anti-oxidant, anti-atherosclerotic and anti-inflammatory activities (Mukund et al., 2017; Sharifi-Rad et al., 2021). 25, 50 mg/kg *genistein* significantly inhibited NF-κB signaling in schistosome-infected liver tissues, as evidenced by decreased mRNA levels of MCP1, TNFα, and IL10, and decreased expression of TGF-β1 and α-SMA (Wan et al., 2017). The same concentration of *genistein* reversed the reduction of STRT1 expression and activity in schistosome liver fibrosis tissues. By suppressing SIRT1 activity, 5–10 and 20 µM doses of *genistein* significantly decreased HSC-T6 cell activation (Zhou C. et al., 2021). Previous studies have demonstrated that *genistein* attenuates hepatic fibrosis by inhibiting TGF-β/Smad signaling through downregulation of p-Smad3 (Ganai and Husain, 2017). Interestingly, the knockdown of SIRT1 enhanced TGF-β1-induced Smad3 phosphorylation (Ma et al., 2019). Thus, *genistein* may also ameliorate schistosomiasis liver fibrosis via the SIRT1/TGFβ/Smad3 pathway. The above studies suggest that *genistein* may be an effective drug for the treatment of schistosomiasis liver fibrosis.

#### 3.1.7 Paeoniflorin

*Paeoniflorin* is the main active ingredient of the *Paeonia lactiflora Pall.*, a monoterpene glycoside compound used in the treatment of cancer, depression, diabetes, liver disease, and autoimmune disorders (Ma et al., 2020; Zhang and Wei, 2020). Mouse peritoneal macrophages that are stimulated to produce TGF-β1 by SEA, causing the promotion of the proliferation of HSC and the synthesis of collagen. Chu et al. demonstrated for the first time that 7.5–120 mg/L of *paeoniflorin* (colchicine, positive control, 1 µM) selectively downregulated the level of Smad3 phosphorylation through TGF-β1 signaling and inhibited the proliferation of HSC(Chu et al., 2007). Another study found that 30 mg/kg *paeoniflorin* significantly reduced schistosome-induced elevated levels of IL-13 and decreased STAT6 phosphorylation levels and collagen I expression by increasing SOCS-1 expression (Li et al., 2010). Further studies showed that 100 μg/mL *paeoniflorin* directly or indirectly inhibited alternative activation of Kupffer cells by reducing JAK2 and STAT6 phosphorylation (Chu et al., 2011). *Paeoniflorin* may be a promising drug for the treatment of fibrosis in schistosomiasis.

### 3.2 Plant extracts

The roots, stems, leaves, flowers, and fruits of plants are processed using certain technological methods to obtain herbal bioactive ingredients that affect diseases.

#### 3.2.1 Silymarin

*Silymarin* is a standardized dried extract of the fruit and seeds of *Silybum marianum (L.) Gaertn*. *Silybin, isosilybin, silydianin and silychristin*, are the four major flavonoid lignan isomers in *silymarin*. *Silybin* is the main active ingredient and is known for its anti-inflammatory, antioxidant, anti-fibrotic, and hepatoprotective effects (Abenavoli et al., 2018; Gillessen and Schmidt, 2020). Mata-Santos et al. found that *silymarin* (*silybin* content,47%) reduced the size of liver granulomas and alleviated liver fibrosis by inhibiting the production of pro-inflammatory and fibrotic factors, including IL-13, IL-4, TNF-α and TGF-β1, and HSC proliferation (Mata-Santos et al., 2010; Mata-Santos et al., 2014; El-Sayed et al., 2016). In a study of acute and chronic schistosomiasis liver fibrosis, *silymarin* (750 mg/kg/day, 5 days/week for 6 weeks) significantly reduced hepatic HYP levels, TGF-β1 and MMP-2 expression and restored GSH levels in both stages, reducing hepatic egg load and regulating granuloma size (El-Lakkany et al., 2012).

#### 3.2.2 Green tea extract

*Camellia sinensis (L.) Kuntze* is a perennial woody plant whose young leaves and flowers are processed into beverages or medicines (Butt et al., 2015). Green tea has been widely demonstrated to have preventive effects against diabetes, cancer, and cardiovascular disease. Its health properties are attributed to the bioactive polyphenols contained in it, particularly catechins (Xing et al., 2019). Epigallocatechin gallate contains 30%–50% of green tea catechins and is known for its potent antioxidant, anti-obesity, anti-inflammatory, anti-cancer, and other pharmacological activities (Yang et al., 2020). Bin Dajem et al. showed that green tea at a concentration of 3% (w/v) reduced hepatocellular necrosis and perivascular collagen fibers by decreasing lipid peroxidation, but failed to significantly improve liver function (Bin Dajem et al., 2011). Another study found Matcha (a Japanese green tea powder made from finely powdered dried tea leaves) to have lower levels of polyphenols and higher levels of caffeine, quercetin, and rutin than traditional green tea (Ramez et al., 2021). Matcha (3 g/kg b. w) contained more theanine and rutin than other green teas, reducing TNF-α, IFN-γ, and IL-13 levels, increasing IL10 levels, which led to the inhibition of the development of liver granulomas, the restoration of SOD, CAT and GSH-Px activity as well as MDA and TAC levels through antioxidant capacity (Kochman et al., 2020). The natural components of green tea may be a promising complementary treatment therapy for schistosomiasis.

#### 3.2.3 Boswellia serrata resin extract

*Frankincense* is the resin that exudes from the bark of the *Boswellia Roxb.* tree, a member of the olive family, and boswellic acid is the most important triterpenoid of *frankincense*, especially 3-O-acetyl-11-keto-β-boswellic acid (β-AKBA) (Al-Harrasi et al., 2021). Liu et al. combined *frankincense* oil resin extract with cyclodextrin (BSE-CD) to address hydrophilicity issues. They first found that liver egg granulomas formed by the eggs of *S. japonicum* contained high levels of leukotriene B_4_. BSE-CD (280 mg/kg for 3 weeks) significantly reduced the size of liver granulomas, possibly caused by the reduced expression of MMP-9, LTB_4_, and PGE_2_ (Liu M. et al., 2013). Further postulated mechanisms suggested that it could reduce the inflammatory response around eggs by inhibiting NF-κB signaling and reducing the expression of VEGF, TNF-α, and MCP-1 in mice (Liu et al., 2014).

#### 3.2.4 Ampelopsis grossedentata extract

*Ampelopsis grossedentata (Hand. -Mazz.) W.T. Wang*, also known as vine tea, is a plant of the genus Ampelopsis in the family Vitaceae, mainly distributed in southern China. The pharmacological effects of vine tea are mainly summarized as anti-inflammatory and analgesic, hepatoprotective, hypotensive, hypolipidemic, antitumor, and anti-aging (Xie et al., 2019; Tong et al., 2020; Wang et al., 2023). Flavonoids are the main efficacy components of vine tea, which includes *dihydromyricetin, myricitrin, myricetin, quercetin, rutin,* and *kaempferol* (Xu et al., 2012). *Dihydromyricetin* has the largest content with a mass fraction of 34% and is considered to be foundational for the health benefits of vine tea (Feng et al., 2018; Zhang Q. et al., 2021). The total flavonoids of vine tea have been proven to have anti-liver fibrosis effects (Li et al., 2022). *Ampelopsis grossedentata* extract containing 90% *dihydromyricetin* (150 mg/kg for 8 weeks) significantly ameliorated hepatic fibrosis in *S. japonicum*-infected mice, which was superior to praziquantel alone. (Fang et al., 2010). 30 μM *Dihydromyricetin* significantly inhibited the activation of HSC-T6 cells *in vitro*, mediated through the promotion of AMPK phosphorylation and inhibition of the TGF-β1/Smad signaling pathway (Zhang et al., 2018). *In vivo*, it (100, 200, 400 mg/kg) downregulated TGF-β1/Smad signaling, improved liver function and reduced ECM deposition. Colchicine was used as a positive control at a dose of 0.2 mg/kg (Liang et al., 2019). In addition, another active ingredient, *myricetin* was shown to have a toxic effect on *S. japonicum* worms through induction of apoptosis, with an LC50 of 600 μM at 24 h. Interestingly, it (at 250 mg/kg) reduces the number of worms and eggs as well as the size of liver granulomas by modulating the immune response (lowering the ratio of Th2 and Th17 cells) (Huang et al., 2020).

#### 3.2.5 Ginger extract

*Ginger* (*Zingiber officinale Roscoe*), a perennial herb of the *ginger* family, is a medicinal plant with the same origin as food, and has the effect of promoting sweating and relieving symptoms, causing a warming sensation of the body, and suppresses vomiting (Zhang M. et al., 2021). *Ginger* crude aqueous extract (500 mg/kg) slowed the development of granulomatous inflammatory infiltrates and reduced hepatic egg load after schistosome infection, which was more pronounced after treatment with *ginger*-derived nanoparticles (Mostafa et al., 2011; Abd El Wahab et al., 2021). This may be related to the powerful antioxidant effect of *ginger* extract and its ability to scavenge free radicals, as evidenced by the restoration of CAT activity and MDA levels. Another study showed that ethanolic extract of *ginger* also inhibited oxidative stress and inflammatory mediators to improve schistosomiasis-associated liver fibrosis (Aly and Mantawy, 2013). Interestingly, Sanderson et al. suggested that the ethyl acetate extract of *ginger* (150 mg/kg) did not kill the egg load and helminth load of schistosome-infected mice, which was attributed to the alternative extraction solvent and the varying treatment doses of the extracts (Sanderson et al., 2002). Given the lack of data on the role of *ginger* extract as a treatment for schistosomiasis-associated liver fibrosis, further study is required.

#### 3.2.6 Other extracts

*Ziziphus spina-christi* leaf extract (ZLE) is extracted from *Z. spina-christi (L.) Willd*, alkaloids and flavonoids are the main constituent classes. The pharmacological effects of it include antibacterial, anti-inflammatory, antiparasitic, and anticancer (Abdulrahman et al., 2022). 600 mg/kg Ziziphus spina-christi showed granuloma reduction and anti-hepatic fibrosis in mice infected with *S. haematobium* (Alghamdi et al., 2023). 400 mg/kg ZLE treatment reduces hepatic granuloma area in mice infected with *S. mansoni* and reduces hepatic fibrosis by inhibiting the expression of TGF-β1, VEGF, α-SMA, TIMP-1, and MMP-9, as well as inhibiting oxidative stress and inflammation by upregulating Nrf2 (Almeer et al., 2018). An aqueous extract of *Moringa Oleifera Lam.* Leaves (150 mg/kg for 15 days) significantly reduced NF-κB expression and thereby ameliorated schistosome-induced hepatic fibrosis (Saad El-Din et al., 2023). Ceratonia siliqua pod extract (*Ceratonia siliqua L.*) at doses of 300 mg/kg or 600 mg/kg reduced the area of granulomas and fibrosis by counteracting oxidative stress and decreasing TIMP-2 expression (Al-Olayan et al., 2016). 1.5 g/kg Artichoke leaf extract (*Cynara scolymus L.*) reduces granuloma size by increasing HSC recruitment within the granuloma (Sharaf El-Deen et al., 2017).

## 4 Conclusion

The main objective of this paper is to summarize the mechanistic studies of selected metabolites and plant extracts for the treatment of schistosomiasis-associated liver fibrosis. Some metabolites or plant extracts that did not address pharmacological mechanisms or were partially uncommon were excluded from the review. In addition, in the literature reviewed reported inconsistent ranges in dosage and varying assessment criteria for fibrosis.

**FIGURE 1:**
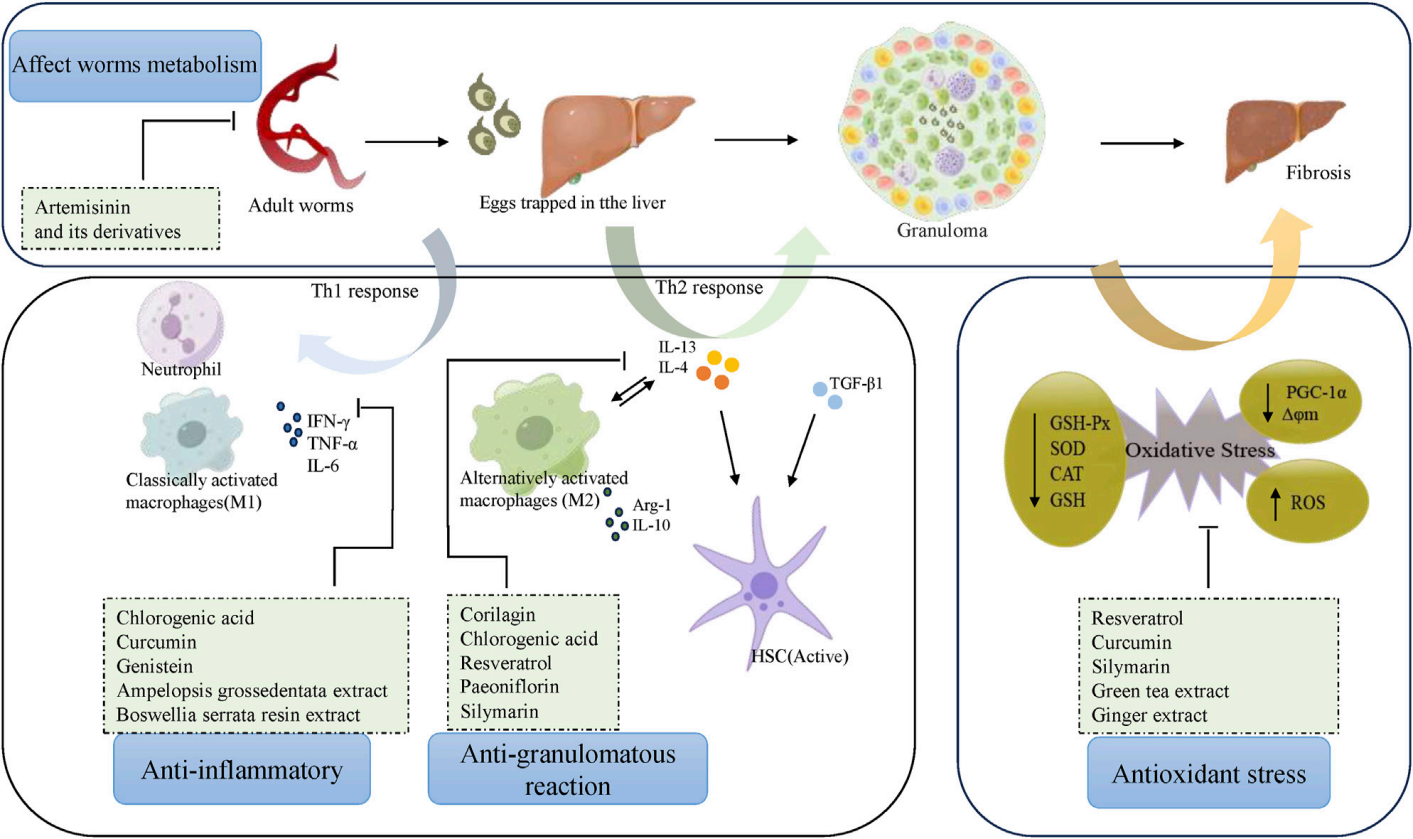
Pharmacologic mechanisms of natural products against schistosomiasis-associated liver fibrosis.

Praziquantel is currently the most commonly used medication for schistosome prophylaxis and treatment. Due to praziquantel’s low toxicity to eggs, it is not effective in preventing the progression of liver fibrosis caused by schistosomiasis infection According to pharmacological monographs, current literature, and experimental studies, metabolites or plant extracts have the potential to treat schistosome-induced liver fibrosis. Their therapeutic mechanisms are characterized by 1) reduction of inflammation, 2) reduction of the granulomatous response, 3) reduction of oxidative stress, and 4) reduction of egg loading (Figure 1). There is considerable evidence that therapeutic efficiency is improved by combining metabolites/plant extracts with praziquantel, or with nanocarriers, or by using liposomes. Previous basic and clinical studies have shown good safety and tolerability for metabolic substances like resveratrol and chlorogenic acid, but adverse effects cannot be excluded due to experimental variability, inter-individual variability, and the lack of clinical trial reports. Furthermore, studies on the above metabolites and botanicals for the treatment of hepatic fibrosis due to schistosomiasis have been limited to basic research and no clinical studies have been reported. In conclusion, the protective role of natural products in the treatment of liver fibrosis in schistosomiasis needs to be confirmed by more standardized cellular studies, and supported by *in vivo* data from animal studies, which if promising should be escalated to randomized controlled clinical trials in humans.
